# Formulation of Entomopathogenic Nematodes for Above-Ground Use Against Tomato Leaf Miner, *Phthorimaea absoluta*

**DOI:** 10.3390/insects16020189

**Published:** 2025-02-10

**Authors:** Bancy W. Waweru, Joelle N. Kajuga, Athanase Hategekimana, Assinapol Ndereyimana, Lydie Kankundiye, Christine Umulisa, Alphonse Nyombayire, Marie Mutumwinka, Primitive M. Ishimwe, Didace Bazagwira, Grace C. Mukundiyabo, Vincent de Paul Bigirimana, Xun Yan, Jozsef Kiss, Stefan Toepfer

**Affiliations:** 1 Rwanda Agriculture and Animal Resources Development Board, Huye P.O. Box 5016, Rwanda; joellekajuga@gmail.com (J.N.K.); athanase.hategekimana@rab.gov.rw (A.H.); assinapol.ndereyimana@rab.gov.rw (A.N.); lydie.kankundiye@rab.gov.rw (L.K.); christine.umulisa@rab.gov.rw (C.U.); alphonsenyombayire@yahoo.com (A.N.); mmutumwinka17@gmail.com (M.M.); mupillia0@gmail.com (P.M.I.); bazagwiradidace@gmail.com (D.B.); 2 AgroPy Ltd., Musanze P.O. Box 81, Rwanda; cgrace@agropyltd.com; 3 Department of Crop Sciences, School of Agriculture and Food Sciences, College of Agriculture, Animal Sciences and Veterinary Medicine, University of Rwanda, Musanze P.O. Box 210, Rwanda; bigirimanavp@gmail.com; 4 Key Laboratory of Green Prevention and Control on Fruits and Vegetables in South China, Ministry of Agriculture and Rural Affairs, Zhongkai University of Agriculture and Engineering, Guangzhou 510225, China; yanxun@zhku.edu.cn; 5 Department of Integrated Plant Protection, Plant Protection Institute, Hungarian University of Agriculture and Life Sciences MATE, 2100 Godollo, Hungary; s.toepfer@cabi.org; 6 CABI, 2800 Delemont, Switzerland

**Keywords:** biological control, pest management efficacy, *Steinernema carpocapsae* RW14-G-R3a-2, East Africa, invasive alien species

## Abstract

The tomato leaf miner *Phthorimaea* (syn. *Tuta*) *absoluta* Meyrick (Lepidoptera: Gelechiidae) is invasive in many agricultural regions. Its larvae feed inside leaf mines or tomato fruits and are difficult to reach with plant protection products. In contrast, entomopathogenic nematodes (EPNs) are highly virulent and can search for larvae. The challenge is to formulate EPNs to remain protected on a sprayed leaf surface until they enter the mines. We tested 11 formulations, including 2 oils, 4 gels or thickeners, 2 surfactants, 2 UV protectants, water, and combinations with and without the EPN *Steinernema carpocapsae* (Weiser) RW14-G-R3a-2 (Rhabditida: Steinernematidae), under non-closed low-humidity conditions. Six tomato leaf experiments with 1000 EPNs sprayed per leaf showed that EPNs in 0.25 to 0.5% canola oil or in 5% alkyl polyglycoside surfactant were the most effective (26 to 37%). Other formulations and an insecticide had less or no effect. Seven other bioassays showed that most formulations did not adversely affect EPN survival or pathogenicity. We assume that formulations of EPNs can be further improved, such as with other flowable gels or combinations, and encourage investment in the development of practical and economic protective sprayable formulations of EPNs against leaf miners to reduce reliance on chemical insecticides.

## Simple Summary

The immature stages of the tomato leaf miner moth are larvae that feed inside tomato leaves and fruits. This pest insect is scientifically called *Phthorimaea absoluta.* It is of American origin but has invaded many agricultural regions worldwide. Management with pesticides is difficult because the larvae are hidden inside the leaves. Entomopathogenic nematodes are tiny worms that are used as biocontrol agents of insect pests, because they can infect insect larvae and multiply in them. They can also search for larvae inside the leaves, which is an advantage over pesticides. The challenge is to formulate nematodes so that they remain protected on a sprayed leaf surface until they enter the leaves. We tested two oils, four thickeners, two surfactants, two UV protectants and water with and without a nematode called *Steinernema carpocapsae*. In our experiments, we sprayed infested tomato leaves with 1000 formulated nematodes. The results showed that nematodes in 0.25 to 0.5% canola oil or in 5% surfactant were the most effective. Other experiments showed that most formulations did not adversely affect the nematodes themselves. We assume that the formulation of nematodes can be further improved. We encourage investment in the development of protective sprayable formulations of nematodes to reduce reliance on chemical insecticides.

## 1. Introduction

Tomato, *Solanum lycopersicum* (L.) (Solanales: Solanaceae), is an important cash crop for farmers. Tomato is an important part of the diet of consumers across the globe. *Phthorimaea* (syn. *Tuta*) *absoluta* Meyrick (Lepidoptera: Gelechiidae) is an invasive pest of solanaceous crops. It is native to South America and has invaded large areas of Africa, Europe, and Asia [1]. The larvae burrow through the tomato leaves but also attack the fruits. The latter has a serious impact on crops, causing high yield and quality losses [2,3].

Tomato leaf miners are difficult to control because they feed inside leaves and fruits and are therefore largely protected from foliar sprays, regardless of being synthetic insecticide treatments or microbial biopesticides [4]. Moreover, spraying pesticides during tomato flowering, which coincides with fruit set and often with *Ph. absoluta* attack, is problematic because of the risk to affect pollinators. On the other hand, spraying on tomatoes before harvest is restricted by pre-harvest intervals. If farmers nevertheless spray pesticides to protect the tomato harvest, this can pose a serious risk to consumers [5,6]. Safer pest management tools are urgently needed to reduce risks to workers, consumers, and the environment [7].

Entomopathogenic nematodes (EPNs) could be a preferred solution, as they are among the few nature-based agents, besides parasitoids and predators, that can actively search for the target [8,9]. Many EPN species and strains are known to be highly pathogenic to the larvae of *Ph. absoluta* [10,11,12,13,14]. This is probably because the above-ground larvae and the usually soil-inhabiting EPNs have not co-evolved [15,16,17]. EPNs do not leave residues on the crop, and pose no risk to farmers, consumers, the crop, pollinators, or the environment [18].

The difficulty, however, is that EPNs are sensitive to desiccation and to UV radiation [19]. Therefore, sprayed EPNs may die before reaching and attacking the pest. Therefore, their above-ground application, especially on plant surfaces, is challenging [3,20]. It is hypothesized that EPNs sprayed onto foliar surfaces would take 10 min up to one hour to enter the mines where pest larvae could be attacked in their hidden environment [B. Vandenbossche, 2023, pers. comm.] [10].

Although EPNs are successfully used above ground in protected areas such tree barks or leaf whorls [21], spraying across plants foliage is rare. This is with the exception of highly humid environments, such as irrigated golf courses or dense leafy vegetables in greenhouses [22,23]. For the treatment of tomato leaves, EPNs would need to be formulated in a way that ensures leaf surfaces are covered by the sprayed EPN solution and would not dry out within an hour. At the same time, the used formulations would need, despite retaining water, to still allow the movement of EPNs towards and into the leaf mines [10,24].

Recent advancements in EPN formulations have yielded promising results, with various species of *Steinernema* and *Heterorhabditis* being formulated in a range of delivery formats, including wettable powders, water-dispersible granules, alginate beads, surfactants, and oil emulsions [25,26,27,28,29,30]. These formulations have been utilized in targeting diverse insect pests. However, for *Ph. absoluta,* few experimental studies have focused on formulating EPNs to increase their efficacy in managing the insect [11,20]. For example, Ben Husin [20] demonstrated that the addition of 1% xanthan gum or 1.5% liquid fire gel concentrate can augment the efficacy of EPNs against *Ph. absoluta* larvae. However, it should be noted that the majority of these experiments were conducted under conditions of artificially high humidity, which may not always reflect real agricultural conditions.

Building on the few existing studies, we aimed to further develop suitable EPN formulations against *Ph. absoluta* larvae. Specifically, the present study aimed to (i) test a number of under-tested formulations for their suitability for application to tomato leaves against the larvae, and (ii) investigate whether the different formulations have any negative effect on the EPNs themselves, namely, on their survival and pathogenicity. It is hoped that the findings will pave the way for the development of a commercial EPN-based biocontrol solution for use against this devastating tomato pest in an IPM or organic farming context.

## 2. Materials and Methods

### 2.1. Target Insect

The target insects in this study were larvae of *Phthorimaea* (synonym *Tuta*) *absoluta* Meyrick (Lepidoptera: Gelechiidae). Tomato leaves with mined leaflets containing living larvae were collected from heavily infested tomato plants. In total, 50 to 60 litres of infested leaves were collected for each experiment and then transported to the laboratory.

**Figure 1:**
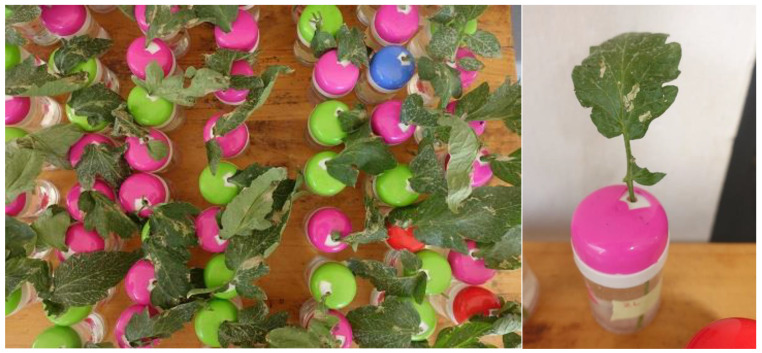
Experimental setup with *Phthorimaea absoluta*—infested tomato leaves placed into cylinders with water. Formulated entomopathogenic nematodes were sprayed onto both sides of the leaflets using handheld sprayers. Open-leaf conditions were maintained without extra moistening of the environment to simulate realistic conditions (Photo S. Toepfer).

At the start of each experiment, the number of living larvae was determined per leaflet by checking tunnels and leaf liaisons under light, counting moving larvae or larvae that started moving after being touched with a pencil. The pencil did not harm the larvae due to its round, non-sharp tip. Single leaves with 1 to 10 mines with larvae across the leaflets were cut and used (average of 2.7 larvae per leaf, ±2.5 SD). They were of different sizes and ages. Each leaf was placed into a cylinder with water for experimentation (Figure 1).

### 2.2. Target Crop

The target crop was *Solanum lycopersicum* (L.) (var. Shanty and var. EVA F1, Solanales: Solanaceae). Experiments were conducted with infested leaves collected from greenhouses of tomato producers around Kicukiru and Bugesera districts in Rwanda. The tomato plants had been grown in rows in the greenhouse soil and had been irrigated every other day. The tomato plants were at their late vegetative to harvesting stage when the infested leaves were cut and used for experimentation.

### 2.3. Entomopathogenic Nematodes

The *Steinernema carpocapsae* strain (Weiser) RW14-G-R3a-2 (Rhabditida: Steinernematidae) was used in the experiments (collection of RAB, from Gakenke district in Rwanda) [31]. Several strains of this EPN species have been reported as highly pathogenic to the larvae of *Ph. absoluta* [10], including the strain used in the presented experiments [12]. The EPNs were reared in vivo on the larvae of *Galleria mellonella* (Lepidoptera: Pyralidae) following the methods of [25,32]. Briefly, 10 larvae were infected with 50 to 100 infective juveniles (IJs) of EPNs on a moist filter paper in a 11 cm Petri-dish. After four days, killed non-decomposed, non-stinky larvae were transferred to a dry filter paper on the island of a small inverted 3 cm Petri dish lid inside a new 11 cm Petri dish [32]. After 7 to 10 days, a 2 mL layer of sterile tap water was added to the base dish, also moistening the filter paper where the dead larvae had been placed. The IJs exited the cadavers within a few hours and were harvested into water and stored in tissue culture flasks (250 mL) under cool conditions at 16 °C for no more than one month until use. Different batches of EPNs were used for the experiments.

### 2.4. Assessing the Efficacy of Differently Formulated EPNs Sprayed onto Infested Tomato Leaves

Six experiments were conducted between September 2022 and February 2023. Those leaf bioassays were conducted in a large laboratory room of the Biocontrol Facility of RAB in Rubona of Huye district in Rwanda (Figure 1). We chose not to use closed-arena bioassay methods because the conditions for EPNs would be too favorable, making it difficult to assess the importance of formulations. Instead, this study simulated natural conditions through a free-air non-closed tomato leaf setup, under relatively low humidity and temperature typical for daytime field conditions (r.h. 40 to 60%, 22 to 25 °C).

Specifically, single tomato leaves with living larvae were cut, and their stems were placed into water through a hole in the lid of a small water-containing cylinder (toothpick cylinders, 4 cm in diameter, 7 cm in height) (Figure 1). The tomato leaflets of a leaf were approximately 3 × 8 cm in area.

The treatments were sprayed as liquid foliar spays onto the upper and lower sides of leaves from a 10 cm distance, using handheld sprayer bottles (200 mL precision sprayer bottles, Chstarina Shenzhen Jingyiteng Electronic Technology Ltd., Shenzhen, China). In total, 11 different formulations were assessed, namely, 2 oils, 4 gels or thickeners, 2 surfactants, 2 protectants and water, as well as some combinations (Table 1). Most of them were tested at various dosages. Water and the commercial botanical insecticide pyrethrin served as negative and positive control without EPNs, respectively. Each formulation and dose were tested in at least four repetitions. A 1.5% carboxymethyl cellulose solution was difficult to spray because it was too viscous. Glucopon + zeolite coagulated after some time.

EPNs were diluted in the formulations, except for positive and negative controls, at a dose of 500 IJs per ml. For each treatment, 0.1 to 0.15 L was prepared and used within an hour. A total of 1000 IJs were sprayed with handheld sprayers in 2 mL of formulations on each leaf, which is a low dose. Each treatment was applied onto 10 leaves per experiment, leading to a samples size of 40 to 60 leaves per treatment, depending on the combination and availability of leaves.

The mortality of *Ph. absoluta* larvae was assessed 2, 4, and 6 days after treatment. The natural background mortality of larvae was 0.5 ± 2.1% SD, which indicates a good quality of bioassays having an acceptable variability between experimental replicates and being far below the 10% standard threshold of natural mortality in insect assays [33]. Some leaves dried out during the experiments, and such data were discarded.

### 2.5. Assessing the Effects of Formulations on EPNs

To assess the potential negative effects of spray formulations on EPNs, 60 mL of 10 different formulations were prepared with approximately 30,000 IJs each (Table 1). Seven experimental replicates were implemented, i.e., with seven different production batches of the EPNs. EPNs were incubated with their formulation at 16 °C for three days.

The mortality of IJs was assessed at 24, 48, and 72 h following mixture with the formulations. Six subsamples of 10 μL were taken from each formulation and living and dead IJs were assessed using a stereomicroscope. IJs were considered alive if they exhibited movement, were curved, or had a slight curve at the tip and end. IJs were considered dead if they had begun to decompose, were entirely straight, and/or contained air bubbles inside their bodies. After a 72 h period, subsamples of 500 IJs in 1 mL were taken from each formulation and sprayed onto moist filter paper with six *G. mellonella* larvae to assess EPN pathogenicity. Larval mortality was assessed after 3 and 5 days.

### 2.6. Data Analysis

Data on the effects of treatments were normalized to the data of the corresponding negative control to reduce the potential effects of EPN batches and other potential variations between experiments. This allowed the pooling of data across experiments. Distributions of data were investigated for normality using histograms as well as normal and detrended normal probability Q–Q plots [34]. Also, the skewness and kurtosis of the residuals were observed for normality. Equality of variances was assessed using Levene’s test. Data appeared normally distributed, and no data transformation was needed. Therefore, the effects of treatments were analyzed through a one-way ANOVA and multiple comparisons, using the Tukey HSD post hoc comparison of data with equal variances and the Games–Howell post hoc comparison with unequal variances. The statistical software IPM SPPS 13 was used [34].

## 3. Results

### 3.1. Efficacy of Differently Formulated EPNs Sprayed onto Infested Tomato Leaves

**Figure 2:**
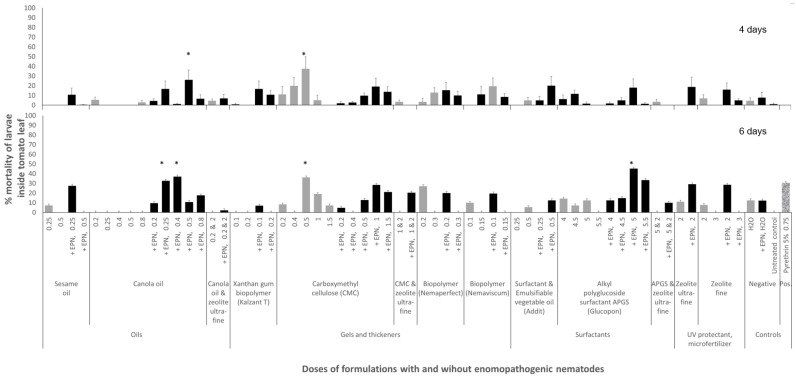
Mortality of *Phthorimaea absoluta* larvae mining inside tomato leaves as a result of treatments by different spray formulations with and without the entomopathogenic nematode (EPN) *Steinernema carpocapsae* RW14-G-R3a-2. Light gray bars represent data from formulations alone, and black bars from formulations with EPNs. A total of 1000 EPN were sprayed in 2 mL of formulations per leaf. At least 4 repetitions were performed for each formulation and dose. Formulation doses are given in ml or g of formulation per 100 mL of water. Error bars represent the standard error of the mean. Stars on bars indicate differences from the negative controls at *p* < 0.05 according to the Games–Howell post hoc multiple comparison test following one-way ANOVA.

Formulated EPNs were able to reduce the larvae of *Ph. absoluta* in tomato leaves. This was observed within 4 days (ANOVA: F_63;962_ = 2.4, *p* < 0.0001, adjusted R^2^ = 0.14), as well as within 6 days (F_63;584_ = 1.5, *p* = 0.006, R^2^ = 0.15) (Figure 2). Regardless of their formulation, EPNs did not kill the larvae rapidly within 2 days post-treatment (F_63;1086_ = 1.2, *p* = 0.18, R^2^ = 0.01).

Among the 11 tested formulations or their combinations, EPNs in canola oil (e.g., at 0.25 to 0.5 mL/0.1 L) or in an alkyl polyglycoside surfactant (Glucopon 5 g/0.1 L) appeared most effective. EPNs in other formulations either did not control the larvae or only provided minimal control.

**Figure 3:**
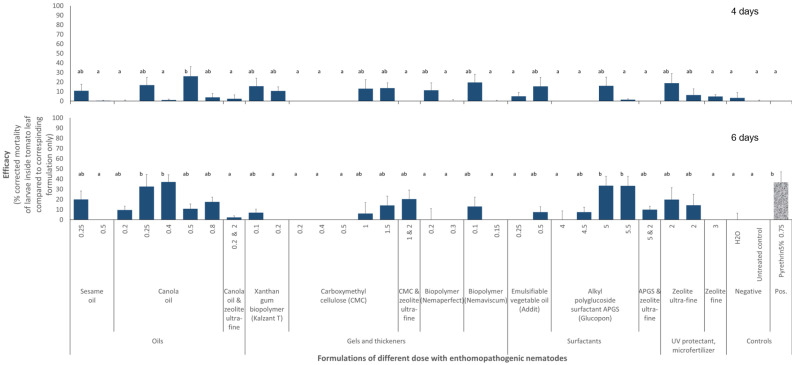
Relative efficacy of formulations of the entomopathogenic nematode (EPN) *Steinernema carpocapsae* strain RW14-G-R3a-2 sprayed against the larvae of *Phthorimaea absoluta* mining inside tomato leaves, corrected to their corresponding formulations without EPN. At least 4 repetitions were performed for each formulation and dose. Formulation doses are given in ml or g of formulation per 100 mL of water. Error bars represent the standard error of the mean. Letters on bars indicate differences at *p* < 0.05 according to the Games–Howell post hoc multiple comparison test following one-way ANOVA.

Specifically, EPNs in 0.5% canola oil killed 26 ± 40 SD % of larvae within 4 days. Within 6 days, EPNs in 0.25 or 0.4% canola oil killed 33 ± 47% and 37 ± 29% of larvae, respectively, and EPNs in 5% alkyl polyglycoside surfactant killed 34 ± 36% of larvae within 6 days. Relative control efficacies compared to the formulations alone ranged from 33 to 37% within 6 days (Figure 3).

The botanical insecticide pyrethrin had no immediate effects on the larvae in the mines but reduced 37 ± 41% of them within 6 days.

The formulations themselves did not negatively affect the insect larvae, with the exception of carboxymethyl cellulose at a 0.5% non-gelling concentration (Figure 3). Adding zeolite as an additional UV protectant and carrier to oils or surfactants had no negative or positive effects on the larvae.

### 3.2. Effects of Formulations on EPNs

**Figure 4:**
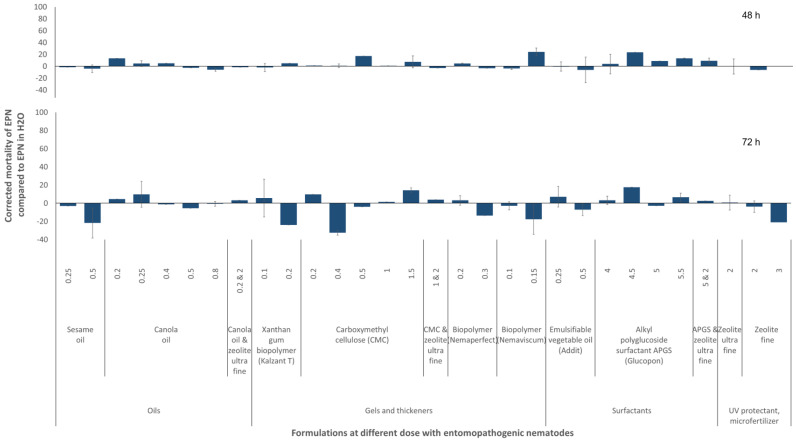
Relative influence of formulations on the survival of the entomopathogenic nematode (EPN) *Steinernema carpocapsae* strain RW14-GR3a-2 in laboratory assays. Relative mortality corrected to the natural background mortality in water. In case of negative mortality compared to the control, this means potential positive effects on nematode survival. Nematodes assessed under stereomicroscope. Six dishes per formulation in each of the seven experiments. Formulation doses are given in ml or g of formulation per 100 mL of water. Error bars represent the standard error of the mean. No statistical differences to the negative control at *p* < 0.05 according to the Games–Howell post hoc multiple comparison test following one-way ANOVA.

Multiple comparison tests revealed that none of the 11 tested formulations, including 2 oils, 4 gels or thickeners, 2 surfactants, 2 UV protectants, or water, as well as their combinations caused any mortality among the EPNs within 2, 24, 48, or 72 h of exposure, regardless of the dose of formulation (Figure 4). Nevertheless, there was a slight formulation x dose effect on EPN mortality (ANOVA “formulation and dose” at *p* < 0.0001 at 24, 48, or 72 h, F_31;405 to 316_ = 5.7, 3.2, 4.4, and adjusted R^2^ = 0.27, 0.14, 0.17, respectively).

**Figure 5:**
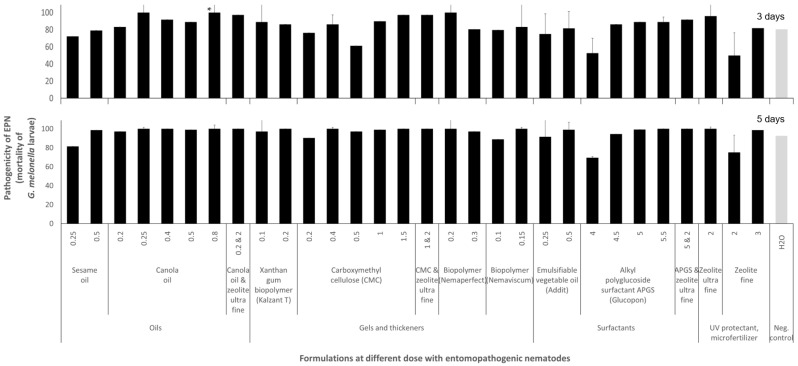
Influence of formulations on the pathogenicity of the entomopathogenic nematode (EPN) *Steinernema carpocapsae* RW14-GR3a-2 in laboratory assays. Six dishes, each containing six *Galleria mellonella* larvae per formulation, were used in each of the seven experiments. Formulation doses are given in ml or g of formulation per 100 mL of water. Error bars represent the standard error of the mean. Stars on bars differences at *p* < 0.05 according to the Games–Howell post hoc multiple comparison test following one-way ANOVA.

The factor “formulation and dose” slightly affected the pathogenicity of the formulated EPNs after 72 h of exposure (*G. mellonella* larvae mortality, 3 days post EPN treatment: F_31;347_ = 4.6; R^2^ = 0.24; *p* = 0.0006; 5 days: F_31;347_ = 2.1; R^2^ = 0.1; *p* = 0.00007) (Figure 5). However, multiple comparison tests could not capture specific positive effects of single formulations and doses on the EPN pathogenicity of *G. mellonella*, except for 0.8% canola oil (*p* = 0.009) on EPNs within 3 days post-treatment.

## 4. Discussion

This study has identified some ways to protect EPNs in a formulation that can be sprayed on tomato leaves using standard sprayer equipment, potentially allowing EPNs to successfully reach and control *Ph. absoluta* larvae within the leaf mines of tomatoes. Although formulations and efficacy may need to be further improved, our findings are already a step forward in managing this serious invasive alien pest of tomatoes in a less disruptive way than chemical insecticides.

Our results suggest that most of the 11 formulations tested, including some oils, gels or thickeners, surfactants, UV protectants, or water, may not provide sufficient protection for the EPNs on tomato leaves during their search for the insect target under open-leaf and low-humidity experimental conditions. For example, we could not confirm the beneficial activity of some of the polymer formulations for EPN treatments as reported by [10]. This may be because most formulations appeared to provide only partial coverage of the leaves and/or to dried out too fast in open-leaf situations. Other reasons for failure might include the possibility that some of the formulations may hinder the movement or orientation of the EPNs, although this remains hypothetical and warrants further investigation in the micro-environment of treated tomato leaves. On a positive note, none of the formulations appeared to be toxic to the EPNs themselves or to affect their pathogenicity, which allows for further studies.

Promisingly, our results showed that EPNs in two types of formulations, namely, canola oil and an alkyl polyglycoside polymeric surfactant, did indeed improve some control efficacies of the pest by 26 to 37% within 4 or 6 days. It needs to be noted that we conducted our experiments under an open-tomato-leaf situation, comparable to conditions that would occur in a greenhouse or field. This is in contrast to other studies in Petri dish-like tomato leaf assays at high humidity [35,36,37,38,39], which lead to a concealed environment where EPNs obviously work well and reach higher control efficacies than reported here. Therefore, we believe that our findings are a good starting point for studying EPNs for realistic conditions.

Moreover, control efficacies of around 30%, as reported here, and if reached at early infestations by *Ph. absoluta*, may be sufficient to push populations below the threshold [39]. Also, the observed time periods of applied EPN efficacies within 4 or 6 days are typical for EPNs, as well as for many other biological agents, which need to first infect and then kill the pest insect [40]. Knock-down effects, such as those reached by some synthetic insecticides, cannot be reached by EPNs, and even after 2 days, as not reported here, they do not seem feasible. However, a period of 4 to 6 days to achieve a control effect is considered fast enough for managing populations of this pest. Finally, these formulated EPNs were more effective than the botanical insecticide pyrethrin. Botanicals, as well as synthetic insecticides, have difficulty reaching and controlling hidden larvae. They are therefore often applied repeatedly and at increased dosages. This can lead to the development of resistance in this tomato pest, as has already been reported for major insecticides in most of the invaded regions worldwide [41,42,43].

As stated above, the two most promising formulations were canola oil and an alkyl polyglycoside surfactant. We are not sure why EPN formulated in canola oil worked well, as no such promising results were found for the sesame oil or vegetable oils tested herein, and in combination with surfactants (Figure 2). From our perspective, it appeared that the oil might not have formed a complete protective layer of EPN formulation mixture across the sprayed tomato leaves. There seems also no positive effect of canola oil on the EPNs themselves, as was suggested for some of the other tested formulations, such as xanthan gum biopolymer gel, and potentially sesame oil, carboxymethyl cellulose gel, or zeolite. Some studies reported that canola oil may in itself be a low-toxicity insecticide [44], but our experiments with this oil alone neither affected *Ph. absoluta* nor the EPNs themselves. Nevertheless, we cannot entirely exclude the possible synergistic effects of canola oil with EPNs. In conclusion, the use of canola oil in formulating EPNs against *Ph. absoluta* may warrant further detailed studies, including combinations of canola oil with emulsifiers and potentially higher doses of this oil. Particularly, observational follow-up studies on the behavior of EPNs immediately after spray are recommended.

Conversely, the enhanced efficacy of EPNs in the presence of alkyl polyglycoside polymeric surfactants in formulating EPNs might be easier to explain. We used a 5% solution, which resulted in a thick spray of EPN solution that formed a thin protective layer across large areas of the leaves. Such positive effects of deposition of EPNs on leaves have been reported from thick polymer formulations, such as arabic and guar gum, alginate, and xanthan [45]. In contrast, 4 or 4.5% alkyl polyglycoside solutions had minimal to no effect, presumably due to their liquidity, which hindered their ability to provide adequate protection to the EPNs on the leaves. Notably, gels such as carboxymethyl cellulose or xanthan gum biopolymer did not enhance the efficacy of EPNs in reaching the insect larvae in the tomato leaves. EPNs in gels have been successfully demonstrated for the above-ground control of *Spodoptera frugiperda*, when applied to leaf whorls [19]. However, these gels are applied in maize as thick gels using syringes or gel pumps with the aim that the gel keeps the EPNs in the leaf whorls. This would be a difficult approach for the application of EPNs onto tomato leaves. We therefore diluted the gels to a just-sprayable concentration for use on tomato leaves. For example, carboxymethyl cellulose must be of less than 1.5% concentration to be still sprayable. However, this approach may not have protected the EPNs well enough in our study or may have hindered them from finding the leaf mines. Nevertheless, further studies may be warranted for gel-like sprays of EPNs in tomato plantations, because gels have generally proven to be successful carriers [19,22], and even biopolymers may be revisited [23].

Regarding the most promising formulations found in our study, the next step would be to evaluate the appropriate concentration of the alkyl polyglycoside surfactant and/or canola oil against *Ph. absoluta* under various tomato growing conditions. This could be in greenhouses or polytunnels as well as in an open-field situation. When using this formulation under open-field conditions, a UV protectant may also be added, such as zeolite [45], which itself may also act on *Ph. absoluta* (although this has not been confirmed in our study), or titanium dioxide [46]. It should be noted that zeolite or other minerals may coagulate with certain formulations, as also shown for the alkyl polyglycoside polymeric surfactant with zeolite in our study. It is suggested that these potential interactions between formulations be tested case by case.

## 5. Conclusions

In conclusion, our study has identified sprayable formulations that can protect EPNs and may allow them to successfully reach and control *Ph. absoluta* larvae within the leaf mines of tomatoes. Namely, canola oil and an alkyl polyglycoside polymeric surfactant indeed improved the control efficacies of the pests by 26 to 37%. However, it appears that there are still some developmental steps to be taken to achieve a more effective, practical, and economical application technique for EPNs against *Ph. absoluta* larvae in tomato plantations. Our results provide a foundation for such developments. Ultimately, we hope that in the future, EPNs will be able to control this serious invasive pest in tomatoes and reduce reliance on insecticides in tomato production.

## Figures and Tables

**Table 1:** Characteristics of formulations of entomopathogenic nematodes applied as foliar sprays onto tomato leaves infested with mining larvae of *Phthorimaea absoluta*; six laboratory leaf bioassays containing at least four repetitions of each formulation and dose, with a sample size of 40 to 60 for each; Rwanda, 2022 and 2023.

Ingredients	Tradename and Company	Product Formulation	Unit	Dose per Litre
**Oils**				
Canola oil (syn. rapeseed oil)	AgroPy Ltd., Musanze, Rwanda	liquid	mL	2, 2.5, 4, 5, 8
Sesame seed oil	AgroPy Ltd., Musanze, Rwanda	liquid	mL	2.5, 5
**Surfactants**				
Undisclosed surfactant + emulsifiable vegetable oil	Addit, Koppert Ltd., Berkel en Rodenrijs, The Netherlands	liquid	mL	2.5, 5,
Alkyl polyglycoside polymeric surfactant	Glucopon, AgroPy Ltd., Musanze, Rwanda	gel	gr	40, 45, 50, 55
**Gels and thickeners**				
Undisclosed biopolymer	Nemaperfect, e-nema Ltd., Schwentinental, Germany	powder	gr	2, 3
Undisclosed biopolymer	Nemaviscum, e-nema Ltd., Schwentinental, Germany	powder	gr	1, 1.5
Carboxymethyl cellulose organic polymer	CMC, Sapore Puro, Gioia group, Torino, Italy	powder	gr	2, 4, 5, 10, 15
Xanthan gum organic polymer	Kelzan T, AgroPy Ltd., Musanze, Rwanda	powder	gr	1, 5
**UV protectant, micro fertilizer**				
Zeolite fine		powder	gr	20, 30
Zeolite ultrafine		powder	gr	20
**Water**				
Water		liquid	mL	1000
**Combinations**				
CMC + zeolite ultrafine	see above	powder	g + g	10 + 20 *
Glucopon + zeolite ultrafine	see above	powder	g + g	50 + 20 *^,+^
CMC + zeolite ultrafine	see above	liquid + powder	mL + g	2 + 20 *
**Positive control**				
Pyrethrin	Pyrethrin 5%, AgroPy Ltd., Musanze, Rwanda	liquid	mL	7.5

* A concentration of 2 g of zeolite ultrafine per 0.1 L is good for zeolite-only sprays, but was reduced to 1 g per 0.1 L for combined formulations. ^+^ This combination is sprayable, but starts to coagulate after some time and may clog nozzles.

## Data Availability

The datasets used and analyzed during the current study are available from the corresponding author upon request.

## Abbreviations

The following abbreviations are used in this manuscript:

EPN	entomopathogenic nematode
ANOVA	analyses of variance
g (mg)	gram (milligram)
L (mL)	litre (millilitre)
